# Structural Relationships between Socio-Cognitive Mindfulness, Everyday Creativity, and Clinical Competence in Nursing Students: Mediating Effects of Everyday Creativity

**DOI:** 10.3390/healthcare12010005

**Published:** 2023-12-19

**Authors:** Mikyoung Lee, Mijung Jung

**Affiliations:** Department of Nursing, Kwangju Women’s University, Gwangju 62396, Republic of Korea; mikylee@kwu.ac.kr

**Keywords:** nursing students, socio-cognitive mindfulness, everyday creativity, clinical competence

## Abstract

Background: Incorporating socio-cognitive mindfulness, which has not received much attention from nursing researchers, this study investigated the structural relationships between socio-cognitive mindfulness, everyday creativity, and clinical competence among nursing students. This study also explored the mediating effect of everyday creativity. Methods: A cross-sectional study was performed with 222 nursing students in South Korea. Students completed the questionnaire evaluating their own socio-cognitive mindfulness, everyday creativity, and clinical competence. Data were analyzed using structural equation modeling and path analysis. Results: Socio-cognitive mindfulness positively influenced everyday creativity (β = 0.791, *p* < 0.01), and everyday creativity also positively influenced clinical competence (β = 0.470, *p* < 0.01). However, the relationship between socio-cognitive mindfulness and clinical competence was not significant. Importantly, everyday creativity fully mediated the relationship between socio-cognitive mindfulness and clinical competence (a × b = 0.372, *p* < 0.01). Conclusions: The results indicate that socio-cognitive mindfulness effectively enhances nursing students’ clinical competence by improving their everyday creativity. The mediating result highlights the significance of everyday creativity in nursing education, underscoring the necessity for training programs aimed at cultivating creativity. This research offers a basis for developing programs that concentrate on socio-cognitive mindfulness and everyday creativity, with the goal of enhancing the clinical competence of nursing students.

## 1. Introduction

Nursing students’ clinical competence is defined as the ability to perform roles proficiently in nursing practice situations through appropriate knowledge, judgment, and skills [1]. Nursing students’ clinical competence is increasingly emphasized as crucial to becoming a professional nurse. To ensure that nursing students graduate with essential nursing competencies and become competent professional nurses, it is necessary to identify various factors that can enhance their clinical competence and incorporate these factors into clinical practicum education [2]. Therefore, nursing researchers are making efforts to elucidate the factors influencing nursing students’ clinical competence; in particular, mindfulness has recently gained attention as a contributing factor to enhancing this ability, and studies are being conducted to explore its roles and functions in improving clinical competence [3,4,5].

Mindfulness is the act of fully focusing on the present moment, being aware, and observing the current experience with complete awareness [6]. Mindfulness is categorized into meditative mindfulness [7] and socio-cognitive mindfulness [8]. Both types of mindfulness share a commonality in accepting the present moment with an open mind and focusing on both external and internal events, so they allow individuals to move away from habitual behaviors and learn new ways of coping with situations [9]. However, they are different in that meditative mindfulness emphasizes non-judgmental focus on the present through meditation [7], while socio-cognitive mindfulness highlights increasing openness to external stimuli, promoting flexible engagement with the environment [10]. Extensive research on meditative mindfulness has been implemented in many disciplines, including nursing [11,12,13], but research on socio-cognitive mindfulness in the nursing field is still in its early stages.

Socio-cognitive mindfulness refers to an active and proactive state of consciousness characterized by a heightened state of immersion and wakefulness; it consists of novelty seeking, novelty producing, flexibility, and engagement [14]. Unlike meditative mindfulness, socio-cognitive mindfulness can yield immediate effects through short-term interventions employing simple and diverse methods, without requiring prolonged meditation trainings; it enhances cognitive flexibility, facilitating smooth adaptation to diverse circumstances [15,16]. Langer [16] suggests that socio-cognitive mindfulness, aiming to enhance cognitive performance and flexibility, may be more applicable to cognitive learning compared to meditative mindfulness. Accordingly, she advocates incorporating socio-cognitive mindfulness into educational settings.

Everyday creativity strengthens individual self-realization and adaptability through creative thinking and inclinations that anyone can exhibit [17,18]. This manifests itself not only in the arts and specialized fields but in all life processes, including everyday activities, and is displayed daily in every task and in diverse places [19]. Therefore, everyday creativity plays a crucial role in an individual’s flexible adaptation and happiness; in addition, it helps people promote self-realization and contribute to the world [17]. With an open-minded attitude that accepts diverse perspectives, everyday creativity is identified as being closely related to socio-cognitive mindfulness, which promotes sub-factors of creativity, such as flexibility and novelty [20,21]. Specifically, individuals can exhibit creativity by moving away from biased automatic thinking through socio-cognitive mindfulness, actively choosing goal-oriented alternatives [22]. 

In fact, previous studies in psychology have shown that everyday creativity was positively associated with socio-cognitive mindfulness [9,20,23] and that creativity and creative learning were promoted through socio-cognitive mindfulness [21]. Furthermore, everyday creativity had a positive influence on student athletes’ performance, and their socio-cognitive mindfulness indirectly affected their performance through everyday creativity [9]. However, there has been scant investigation by nursing researchers into the integration of socio-cognitive mindfulness, everyday creativity, and nursing performance. In a limited study within nursing practice, creativity enables exploring various approaches to issues arising in the nursing field, leading to innovative solutions, and assisting in identifying possible alternatives in the decision-making process [24,25]. That is, creativity is a crucial factor in making effective decisions and performing patient care in diverse situations in clinical settings [26].

Regarding research on socio-cognitive mindfulness in the nursing domain, nursing students’ socio-cognitive mindfulness correlated positively with their positive achievement emotions but correlated negatively with their negative achievement emotions [27,28]. Nursing students’ socio-cognitive mindfulness was also positively related to communication self-efficacy and empathy [29]. Nurses’ socio-cognitive mindfulness had a positive correlation with empathy [30] but a negative correlation with emotional exhaustion [31]. In addition, nurses’ creative energy was beneficial to their clinical competence [24]. Considering these prior findings, it can be inferred that socio-cognitive mindfulness and everyday creativity could be essential psychological factors in enhancing clinical competence among nursing students. Moreover, socio-cognitive mindfulness, which can be relatively easily intervened, is expected to enhance everyday creativity through training and further contribute to improving nursing students’ clinical competence. 

However, despite substantial research on socio-cognitive mindfulness in other disciplines, socio-cognitive mindfulness has not yet received much attention from nursing researchers. Furthermore, despite the significance of socio-cognitive mindfulness, everyday creativity, and clinical competence in nursing [2,26,27,28], studies empirically investigating the influence of socio-cognitive mindfulness and everyday creativity on clinical competence are lacking. Therefore, the present study aims to investigate the structural relationships between socio-cognitive mindfulness, everyday creativity, and clinical competence of nursing students, focusing on socio-cognitive mindfulness at the early stages in nursing research. In particular, this study explores the mediating effect of everyday creativity in the association between socio-cognitive mindfulness and clinical competence. To do this, we propose the following research questions, which have been established based on previous studies:

1. What are the structural relationships between socio-cognitive mindfulness, everyday creativity, and clinical competence among nursing students?
2. Is everyday creativity mediating the relationship between socio-cognitive mindfulness and clinical competence?

## 2. Methods

### 2.1. Research Design

A cross-sectional study was undertaken to examine the relationships between socio-cognitive mindfulness, everyday creativity, and clinical competence among nursing students, while also exploring the mediating effect of everyday creativity. 

### 2.2. Participants and Procedure

This study involved 222 female nursing students enrolled at a women’s university located in a metropolitan city in South Korea. The sample comprised 85 junior students (38.3%) and 137 senior students (61.7%), with an average age of 22.93 (SD = 3.76). The selection of this sample size adhered to the recommendation of Chou and Bentler [32], who suggest that a sample size of 200 or more is ideal when employing maximum likelihood in structural equation modeling (SEM). Thus, the decision to have 222 participants in this study was deemed appropriate for the present research model. Freshmen and sophomores were not included, as they had not yet undergone clinical practicum experiences.

Data were collected online during the period from 1 June to 30 June 2023. Prior to participating, participants were presented with a comprehensive description of the research objectives, ensuring their comprehension of this study’s purpose and significance. Participants were informed of their right to withdraw from this study at any point if they chose to do so. Following this, they voluntarily granted their consent to partake in the research and proceeded to complete a questionnaire encompassing assessments related to socio-cognitive mindfulness, everyday creativity, and clinical competence. To express appreciation for their involvement, a token of gratitude in the form of a five-dollar gift coupon was provided to each participant.

### 2.3. Ethical Considerations

This study received ethical approval from the Institutional Review Board at the university where the participants were enrolled in South Korea (1041465-202302-HR-001-02). All procedures adhered to ethical guidelines for research involving human participants. Participants were guaranteed that their information would be treated with confidentiality and utilized solely for research purposes. They were also informed of their right to withdraw from this study at any point without facing any adverse consequences.

### 2.4. Measures

To measure the degree of socio-cognitive mindfulness, this study used the Korean-adapted Langer Mindfulness Scale (LMS) as validated by Kim [33]. The LMS was initially developed by Bodner and Langer [34], and it includes four aspects of socio-cognitive mindfulness, featuring a total of 21 items: 6 items dealing with novelty seeking, 6 items on novelty producing, 4 items on flexibility, and 5 items on engagement. The responses to these items were measured on a 5-point Likert scale (1: strongly disagree–5: strongly agree), where a higher score indicates a higher level of socio-cognitive mindfulness. The reliability of the original LMS had a Cronbach’s alpha of 0.89 [34], and Cronbach’s alpha was 0.87 in the present study. 

To assess nursing students’ everyday creativity, the Everyday Creativity Scale (ECS) developed by Jeong and Park [17] in Korean was used. The ECS consists of seven dimensions with a total of 36 items: creative flexibility (8 items), alternative problem solving (5 items), adventurous freedom pursuit (5 items), altruistic self-confidence (6 items), relational openness (6 items), distinctive independence (3 items), and explorative immersion (3 items). Participants responded on a 5-point Likert scale (1: strongly disagree–5: strongly agree); a higher score indicates a higher level of everyday creativity. The original ECS exhibited a Cronbach’s alpha of 0.92 [17], while in the present study, the ECS demonstrated a Cronbach’s alpha of 0.91. 

To measure students’ clinical competence, this study used Choi’s [35] modified version of Lee et al.’s [36] clinical competence scale, which was adapted based on Schwirian’s [37] nursing performance scale. Validity and reliability of this scale were well established in previous studies [35,36,37,38]. This scale is comprised of 45 items, encompassing the following five dimensions: nursing process (systematic method for patient assessment, diagnosis, planning, implementation, and evaluation; 11 items), nursing skills (proficiency in technical procedures crucial for safe patient care; 11 items), education/cooperation (role in patient education and collaborative care with healthcare professionals; 8 items), interpersonal relationships/communication (ability to build positive relationships and communicate effectively with patients, families, and colleagues; 6 items), and professional development (commitment to continuous learning, self-improvement, and staying current with evidence-based practices in nursing; 9 items). Responses are rated on a 5-point Likert scale (1: very poor–5: excellent), where a higher score denotes a higher level of clinical competence. The Cronbach’s alpha reliability was 0.92 in Choi [35] and 0.90 in Lee et al. [36]. The Cronbach’s alpha in the present study was 0.95. 

### 2.5. Data Analyses

Data were analyzed using SPSS 28.0 software (IBM Corp., Armonk, NY, USA) and the Mplus 8 program [39]. First, descriptive statistics and correlations between the variables were calculated using SPSS 28.0 software. Second, to investigate the relationships between the main variables of socio-cognitive mindfulness, everyday creativity, and clinical competence, structural equation modeling (SEM) was conducted using the Mplus 8 program. To evaluate the model’s goodness of fit with the data, we considered the following recommended criteria: the comparative fit index (CFI) and the Tucker–Lewis index (TLI), both exceeding 0.90 [40,41], and the root-mean-square-error of approximation (RMSEA) and the standardized root-mean-square-residual (SRMR), both below 0.08 [40,42]. Finally, utilizing Mplus 8, path analysis was performed to explore whether every creativity mediates the relationship between socio-cognitive mindfulness and clinical competence. 

## 3. Results

### 3.1. Preliminary Results

Table 1 presents an overview of the study variables including their means and standard deviations, along with the results of correlation between these variables. All variables exhibited means exceeding the midpoint of the scales (2.5), with clinical competence ranking the highest among them. The mean levels of socio-cognitive mindfulness, everyday creativity, and clinical competence were 3.21 (SD = 0.41), 3.71 (SD = 0.45), and 3.94 (SD = 0.55), respectively. Correlation analysis demonstrated a positive association between socio-cognitive mindfulness and both everyday creativity (r = 0.791, *p* < 0.01) and clinical competence (r = 0.519, *p* < 0.01). Everyday creativity was also positively associated with clinical competence (r = 0.586, *p* < 0.01). 

### 3.2. Relationships between Socio-Cognitive Mindfulness, Everyday Creativity, and Clinical Competence (Research Question 1)

**Figure 1:**
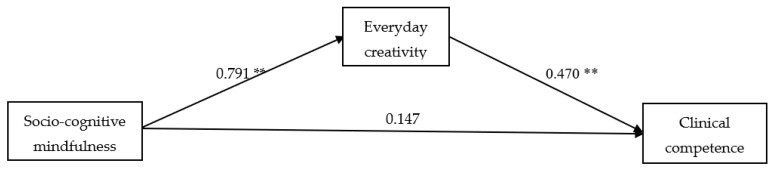
Structural equation model displaying parameter estimates for effects of socio-cognitive mindfulness on everyday creativity and clinical competence. ** *p* < 0.01.

We conducted SEM using Mplus 8 to examine the relationships between socio-cognitive mindfulness, everyday creativity, and clinical competence among nursing students. Figure 1 displays the path coefficients representing the influence of socio-cognitive mindfulness on everyday creativity and clinical competence. This model was saturated, exhibiting a CFI of 1.000, a TLI of 1.000, an RMSEA of 0.000, and an SRMR of 0.000. These fit indices suggest that the data align well with the current model, indicating a perfect fit.

SEM analysis showed a positive influence of socio-cognitive mindfulness on everyday creativity (β = 0.791, *p* < 0.01), as expected. In addition, everyday creativity also had a positive influence on clinical competence (β = 0.470, *p* < 0.01). However, the relationship between socio-cognitive mindfulness and clinical competence did not reach statistical significance. 

### 3.3. Mediating Effects of Everyday Creativity (Research Question 2)

Path analysis with Mplus 8 was conducted to investigate mediating effects of nursing students’ everyday creativity in the relationship between their socio-cognitive mindfulness and clinical competence. We incorporated a bootstrapping analysis, utilizing 1000 resampling iterations, to enhance statistical power and establish statistical significance for both direct and indirect effects. Path analysis revealed that everyday creativity played a mediating role in the relationship between socio-cognitive mindfulness and clinical competence. Table 2 provides details on the total effect, direct effect, and indirect effect (i.e., mediating effect).

Socio-cognitive mindfulness positively influenced everyday creativity (a = 0.791, *p* < 0.01), and everyday creativity positively influenced clinical competence (b = 0.470, *p* < 0.01). The direct effect of socio-cognitive mindfulness on clinical competence exhibited a reduction when controlling for the effect of everyday creativity (c’ = 0.147, ns), as compared to the total effect (c = 0.519, *p* < 0.01). The mediating effect through everyday creativity was significant (a × b = 0.372, *p* < 0.01). This indicates that everyday creativity fully mediates the relationship between socio-cognitive mindfulness and clinical competence among nursing students. 

## 4. Discussion

This study examined the structural relationships between socio-cognitive mindfulness, everyday creativity, and clinical competence among nursing students as well as the mediating effect of everyday creativity in the association between socio-cognitive mindfulness and clinical competence. Structural equation modeling revealed that nursing students’ socio-cognitive mindfulness was positively related to everyday creativity, and that everyday creativity was also positively related to clinical competence; however, the relationship between socio-cognitive mindfulness and clinical competence was not significant. Notably, everyday creativity fully mediated the relationship between socio-cognitive mindfulness and clinical competence. 

Regarding research question 1, first, we found that nursing students’ socio-cognitive mindfulness was positively associated with everyday creativity. This result is consistent with previous findings of a positive correlation between socio-cognitive mindfulness and everyday creativity [9,21,23,43]. To improve creativity, personal characteristics, such as openness and self-confidence; creative thinking skills; and intrinsic motivation are required [44]. Socio-cognitive mindfulness promotes openness by actively exploring new experiences and helps people function more creatively by perceiving negative incidents with diverse directions, which frees them from negative evaluations or comparisons [45]. Creative thinking skills refer to cognitive flexibility, which is associated with divergent thinking through focused attention and exploring alternatives with active attitudes [46]. Socio-cognitive mindfulness helps broaden focused attention through active interaction with the environment, which reinforces cognitive flexibility by forming new categories considering situations and contexts [14]. Moreover, intrinsic motivation is defined as inclinations for pursuing challenges, expanding capacities, exploring, and learning [47]. Individuals with higher socio-cognitive mindfulness explore new knowledge and take unconventional approaches; thus, they would have a stronger intrinsic motivation and demonstrate creative outcomes [48]. Taken all together, nursing students who possess higher socio-cognitive mindfulness will be genuinely engaged with more active attitudes when they learn new clinical nursing skills. These attitudes will lead them to invest efforts to acquire new nursing skills. They will further adapt and refine newly acquired skills to suit their own conditions and characteristics through everyday creativity closely related to socio-cognitive mindfulness [9]. 

Second, everyday creativity was positively related to clinical competence, consistent with earlier findings. For example, one of the strong factors influencing nursing students’ clinical competence was creativity [26], and nurses’ creative energy was helpful in promoting better clinical competence [24]. Creativity enables individuals to equip themselves with both the psychological ability to understand their own personality strengths and the cognitive ability to acquire and utilize knowledge [49]. Furthermore, individuals with creative thinking exhibit unique characteristics that compensate for their weaknesses, such as openness to new experiences, cognitive flexibility, and adventurous tendencies [50]. Based on this, nursing students will be able to acquire new skills and apply them according to their own characteristics, utilizing their everyday creativity. They can exhibit a spirit of challenge, allowing them to complement their weaknesses and reinforce their strengths [9]. In other words, creativity assists nursing students in approaching given tasks with flexible thinking, attempting new approaches from various perspectives. This helps nursing students reconstruct various clinical situations from a new viewpoint, facilitating them to find more beneficial alternatives than traditional methods [26]. This will assist nursing students in making more effective decisions and solving problems creatively in clinical settings, ultimately enhancing their clinical competence [17,25,26].

Third, we discovered that socio-cognitive mindfulness did not have any influence on clinical competence. This was unexpected, considering previous studies that reported a positive correlation between mindfulness and clinical competence [3,5] and students’ athletic performance [51,52]. It is generally believed that individuals with higher socio-cognitive mindfulness can improve their strategies in learning new skills by concentrating on the present and future; thus, they can be equipped with high performance competence [52,53]. Regarding our finding of a non-significant relationship between socio-cognitive mindfulness and clinical competence, it is challenging to offer a detailed discussion here due to the scant research conducted on socio-cognitive mindfulness in nursing. One possible reason could be sub-factors of socio-cognitive mindfulness and nursing students’ learning contexts. This was also pointed out by Yang and Khu [9], a rare study with the same unexpected result among student athletes. LMS, the scale used to measure participants’ socio-cognitive mindfulness in this study, is comprised of novelty producing and flexibility, which are related to the methods to manipulate one’s environment, as well as novelty seeking and engagement, which are related to the level of focus on one’s environment [33]. That is to say, LMS evaluates how students concentrate on their environment and engage proactively in perception, exploration, and decision making [9]. However, nursing students study in a very competitive atmosphere and learn clinical nursing skills following fixed step-by-step procedures. Since they are accustomed to a somewhat rigid learning environment, socio-cognitive mindfulness, which emphasizes exploring and choosing new alternatives from different perspectives with proactive attitudes [16], might have not influenced nursing students’ clinical competence in our study. 

Finally, regarding research question 2, path analysis demonstrated that everyday creativity was fully mediating the relationship between socio-cognitive mindfulness and clinical competence. Specifically, the result presented an indirect positive influence of socio-cognitive mindfulness on clinical competence through everyday creativity. This mediating result is supported by previous findings; for example, the mediating effect of creativity in the association between socio-cognitive mindfulness and job performance [54] and between socio-cognitive mindfulness and sports performance [9]. The mediating result in our study indicates that higher socio-cognitive mindfulness among nursing students is associated with increased everyday creativity, which in turn leads to enhanced clinical competence. The role of everyday creativity as a full mediator suggests that everyday creativity was completely explaining the process by which socio-cognitive mindfulness influenced clinical competence. However, regarding the non-significant direct effect of socio-cognitive mindfulness on everyday creativity, we could not rule out the possibility of a statistical artifact due to the significant correlations between sub-factors of socio-cognitive mindfulness and everyday creativity. To illustrate, socio-cognitive mindfulness originally encompasses the aspect of creativity [44], and there is a potential overlap between the variables in the socio-cognitive mindfulness and everyday creativity questionnaires. This was reflected on the significant relationships between sub-factors of socio-cognitive mindfulness and everyday creativity in our study (r = 0.177–0.670, *p* < 0.01). In addition, we found the significant influence of socio-cognitive mindfulness (β = 0.519, *p* < 0.01) and everyday creativity on clinical competence (β = 0.586, *p* < 0.01) in regression analysis between each variable in our preliminary analysis. Consequently, the significant influence of socio-cognitive mindfulness on clinical competence might have been offset in path analysis. Future studies might investigate this aspect in more detail to elucidate the relationship between these variables.

Nonetheless, this mediating result highlights the significance of everyday creativity in nursing education, underscoring the necessity for training programs aimed at cultivating creativity among nursing students. Given that individuals’ creative achievements can significantly contribute to innovation [55], enhancing creativity in nursing can promote advancements in the field. To enhance nursing students’ creativity, it is essential to first make them aware of the importance of creative thinking, fostering consciousness and attitudes toward creativity. Educational environment and support from various domains are also necessary in this regard [56]. Based on the present finding that socio-cognitive mindfulness positively influenced everyday creativity, to improve student’s creativity, it is recommended that socio-cognitive mindfulness-based programs in the nursing field be developed and implemented.

In general, this research underscores the potential applications and clinical implications of fostering socio-cognitive mindfulness among nursing students. The findings reveal that heightened socio-cognitive mindfulness positively correlates with engaged learning, the acquisition of adaptive skills, and everyday creativity. The capacity of nursing students to approach clinical tasks with adaptable thinking and reconstruct situations from novel viewpoints is influenced by their socio-cognitive mindfulness. This capacity holds promise for improving decision making and creative problem solving in clinical settings. By emphasizing the mediating role of everyday creativity in enhancing the relationship between socio-cognitive mindfulness and clinical competence, this study provides valuable insights. These insights provide a groundwork for future research initiatives and establish a platform for developing educational programs that aim to enhance socio-cognitive mindfulness and creativity among nursing students. This can ultimately contribute to improved clinical competence and innovative practices in nursing.

While our study produced significant results, we recognize certain limitations. First, our study is restricted by including only female students in the sample. Consequently, the generalizability of the findings to both male and female students is limited. To enhance the applicability of our finding to a broader population, future research should aim for more extensive and varied samples, including both genders, thereby facilitating the replication of our findings. Second, the present cross-sectional study from the single source can establish associations between variables, but it is challenging to determine causal relationships and could lead to common method variance. Researchers should consider complementing cross-sectional data with longitudinal studies and multiple data sources in the future to expand our findings. In addition, long-term observations can help establish causality and track changes over time. Third, since data were collected at a single point using self-reported questionnaires, changes over time could not be captured; moreover, self-reported questionnaires limit the depth of information collected, so detailed contexts or nuances of the participants’ behaviors or attitudes might have been missed. Future studies might combine qualitative methods with cross-sectional research to provide a richer understanding of the context, allowing for a comprehensive interpretation of quantitative findings. Finally, there is a possibility that LMS might have not fully captured nursing students’ socio-cognitive mindfulness, considering the non-significant influence of socio-cognitive mindfulness on everyday creativity. LMS was originally developed and validated in Korean using samples of high school students, general university students, and adults. Development of the measure is needed to examine nursing university students’ socio-cognitive mindfulness, reflecting their particular learning environment. With this, the relationship between socio-cognitive mindfulness and other variables could be more objectively investigated in the nursing field. 

## 5. Conclusions

This study delved into the unexplored concept of socio-cognitive mindfulness in nursing, examining its connection with everyday creativity and clinical competence among nursing students. Importantly, this research stands as one of the initial endeavors to uncover the mediating influence of everyday creativity in the relationship between socio-cognitive mindfulness and clinical competence in nursing students. This finding emphasizes the significance of everyday creativity in enhancing clinical competence within the nursing field. Overall, the results suggest that socio-cognitive mindfulness effectively enhances nursing students’ clinical competence by improving their everyday creativity. This study is meaningful in that it has attempted to examine the relationships between socio-cognitive mindfulness, creativity, and clinical competence in nursing, which have barely been explored. By providing information on the relationships between these variables, which have been largely overlooked in nursing literature due to limited prior research, this study can serve as foundational data for future research. Furthermore, this study offers a basis for developing programs that concentrate on socio-cognitive mindfulness and creativity, with the goal of enhancing the clinical competence of nursing students. 

## Figures and Tables

**Table 1:** Correlations, means, and standard deviations for the study variables.

Variables	1	2	3
1. Socio-cognitive mindfulness	1		
2. Everyday creativity	0.791 **	1	
3. Clinical competence	0.519 **	0.586 **	1
Mean ^a^	3.46	3.71	3.94
SD	0.49	0.45	0.55

Note. SD = standard deviation. ^a^ Possible range 1−5. ** *p* < 0.01.

**Table 2:** Everyday creativity as a mediator between socio-cognitive mindfulness and clinical competence.

IV	M	DV			Total Effect	Direct Effect	Indirect Effect
			IV → M (a)	M → DV (b)	IV → DV (c)	IV → DV (c’)	IV → M → DV (a × b)
Socio-cognitive mindfulness	Everyday creativity	Clinical competence	0.791 **	0.470 **	0.519 **	0.147	0.372 **

Note. IV = independent variable; M = mediator; DV = dependent variable. Standardized coefficients are reported. ** *p* < 0.01.

## Data Availability

The data presented in this study are available upon request from the corresponding author.
